# Prehospital predictors of the need for transfusion in patients with major trauma

**DOI:** 10.1007/s00068-022-02132-5

**Published:** 2022-10-12

**Authors:** Holger Gaessler, Matthias Helm, Martin Kulla, Bjoern Hossfeld, Julia Riedel, Juergen Kerschowski, Ingeborg Bretschneider

**Affiliations:** Department of Anaesthesiology, Intensive Care Medicine, Emergency Medicine and Pain Therapy, Federal Armed Forces Hospital Ulm, Oberer Eselsberg 40, 89081 Ulm, Germany

**Keywords:** Trauma, Haemorrhage, Blood transfusion, Blood gas analysis, Emergency medical services

## Abstract

**Purpose:**

Severe haemorrhage is a leading cause of early mortality following major trauma. By early identification of patients at risk, blood transfusion could already be initiated in the prehospital period. Aim of the study was to evaluate the extent to which prehospital lactate and base excess, which are known to be associated with trauma-induced coagulopathy, and additional clinical parameters are associated with the need for early transfusion.

**Methods:**

In this prospective, single-centre observational study, trauma patients treated by a helicopter emergency medical service were included, regardless of the injury severity. Patients with coagulation-influencing drugs in long-term therapy were excluded. Blood samples obtained at the beginning of the prehospital treatment were analysed. Primary outcome was the association of lactate and base excess with the need for early transfusion (resuscitation room or immediate surgery). Receiver operating characteristic curves were created, and the area under the curve (AUROC) was calculated.

**Results:**

Between 2015 and 2018, 21 out of 130 adult trauma patients received blood products during the early in-hospital treatment. Both prehospital lactate and base excess were associated with the transfusion (AUROC 0.731 and 0.798, respectively). The optimal calculated cut-off values were 4 mmol/l (lactate) and − 2.5 mmol/l (base excess). When circulatory instability and suspected relevant bleeding were included, the association further improved (AUROC 0.871 and 0.866, respectively).

**Conclusion:**

Prehospital lactate and base excess could be used in combination with other clinical parameters as indicators of the need for early transfusion. This relationship has yet to be confirmed in the current validation study.

**Trial registration:**

German Clinical Trials Register, www.drks.de (No. DRKS 00009559).

## Introduction

Trauma is the leading cause of death in people under 45 years of age worldwide [1, 2]. In addition to non-survivable injuries, severe traumatic brain injury and massive haemorrhage with bleeding to death are the main causes of death in the early phase after trauma [3, 4]. In particular, haemorrhage accounts for 50% of all deaths in the first 4–6 h [5]. Trauma-induced coagulopathy (TIC), a complication of trauma, further increases the risk of mortality [6].

Given the high mortality risk of haemorrhage, several strategies have been developed to initiate the transfusion of blood and coagulation products in the prehospital period in patients with severe haemorrhagic shock. Initially, these strategies were mainly limited to the military context (e.g. Vietnam, Iraq and Afghanistan) [7]. However, these strategies are also being applied in the civilian setting in Germany and several other European countries, where emergency medical services (EMS) have begun to carry packed red blood cells as well as other blood and coagulation products [8]. Initial structured evaluations have shown an improvement in survival with these strategies [9], and recent subgroup analyses have indicated a survival advantage, especially in cases with long prehospital transportation times [10]. However, the results of recent meta-analyses have been inconsistent [11, 12]. Irrespective of the inconsistencies in the findings, the key to timely transfusion is the identification of patients who are likely to benefit from the transfusion of blood products as early as possible.

Based on earlier recommendations which were mainly oriented towards penetrating trauma, Wang et al. developed the Early Blood Transfusion Needs Score, with which the need for early transfusion can be determined based on the measurements of five parameters collected in the prehospital period [13]. However, in their retrospective study, some of the parameters were measured at the time of hospital admission and not in the prehospital period. Additionally, the different weightings of the values as well as the range of the total score between -4 and 17 indicate that it is not entirely practical for making time-critical prehospital decisions. Furthermore, it is important to consider TIC in the assessment, as the presence of TIC indicates an increased need for transfusion [14]. However, since plasmatic coagulation disorders cannot be detected in the prehospital period due to a lack of diagnostic tools (e.g. thromboelastometry), other parameters that can indicate these disorders must be considered. In a previous study, our research group showed that the blood gas parameters lactate and base excess are associated with TIC and hyperfibrinolysis at the scene of the trauma and can, therefore, serve as indicators of the need for transfusion [15]. Therefore, in the current study, we have used these two blood gas parameters as a basis to create a prediction model of the need for transfusion in the early stage of severe trauma.

The aim of the present study was to, first, investigate to what extent prehospital lactate and base excess values in severely injured patients are associated with the need for early transfusions and, second, in case of a detectable relevant association, create a simple and pragmatic prediction model that includes blood gas values supplemented by clinical parameters for the estimation of an early need for blood transfusion.

## Methods

This study is part of the PREDICT study (Prehospital Evaluation and Detection of Induced Coagulopathy in Trauma), the preliminary results of which have recently been published [15]. The study is registered with the German Clinical Trials Register in Tübingen, Germany (registration no. DRKS 00009559) and has been approved by the Ethics Committee of the University of Ulm, Germany (No. 346/14, Chairman: Prof. Dr. O. Zolk, April 28th 2015). The study was conducted in accordance with the current version of the Declaration of Helsinki. Deferred written consent to participate in the study was obtained from all patients (or their authorised representatives); in case of deceased patients, written consent was obtained from their next of kin.

### Data collection

The PREDICT study was conducted as a monocentric, prospective observational study. Data collection took place between August 2015 and February 2018. Sample size calculation was based on the primary endpoint of the PREDICT study (detection of early coagulopathy after trauma) and is described in detail elsewhere [15]. All injured patients who were treated by the helicopter emergency medical service (HEMS) Christoph 22 and transported to either of the two level I trauma centres in Ulm were included in the study. The restrictions regarding the transport destination were necessary to ensure that complete data on resuscitation room treatment and laboratory results could be obtained and to guarantee the accuracy of the informed consent obtained from the patients. All injured patients treated by the HEMS team could be included (if no exclusion criteria were present), not only those transported by helicopter. In some cases, patients were treated by the HEMS team but transported by ground ambulance (accompanied by the HEMS emergency physician) for operational reasons, e.g. due to short transport times. The treating emergency physicians were exclusively crew members of the HEMS, not of the ground-based EMS. Patterns of injury and severity were deliberately not used as inclusion criteria, to be able to study the whole spectrum of trauma patients.

Patients under 18 years of age and pregnant women were excluded. Since the PREDICT study also aimed to investigate plasmatic coagulation, patients with pre-existing coagulation disorders as well as patients who had already received tranexamic acid before the arrival of the HEMS (and, thus, before the prehospital blood sample was taken) were also excluded.

HEMS Christoph 22, which is based in Ulm, Germany, is staffed by a HEMS physician (specialist/consultant in anaesthesia) and a paramedic. It is deployed within a radius of approximately 70 km, as an EMS only operation and as an addition to ground EMS, e.g. in cases with an expected longer transport route. All patients were treated according to the national and international guidelines [16, 17]. During the study period, no blood or coagulation products and no blood gas analytical equipment were available on the helicopter.

After the arrival of HEMS at the scene of the incident and the start of medical treatment, a venous blood sample was taken from the patient for blood gas analysis (Blood Gas-Monovette^®^: calcium-balanced heparin, 2 ml; Sarstedt, Nuembrecht, Germany). However, in cases that required urgent medical measures, venous access was first established for infusion or administration of medication, and the blood sample was later obtained via a second venous access. The blood sample was transported to the hospital in a thermo-insulated sample container filled with ice water for gas-tight transport. Immediately after hospital admission, blood gas analysis was performed (RAPIDLab^®^ 1265; Siemens Healthineers, Erlangen, Germany). Prehospital vital signs, additional clinical parameters that were assessed, on-scene treatment, and therapy (including infusions and medications) were documented with a form that the HEMS physician filled out after handing over the patient in the hospital.

After admission to the resuscitation room, a new complete blood sample including blood gas analysis was taken. This in-hospital laboratory control (in addition to the clinical impression) was the basis for the decision to administer blood products. In case of ongoing bleeding, the target for haemoglobin was 10 g/dl, otherwise a transfusion trigger at 7–8 g/dl was assumed (depending on known pre-existing conditions, e.g. coronary heart disease) [16, 17]. Coagulation factors were substituted according to the findings of the ROTEM (rotational thromboelastometry) on admission. In all cases, the decision for transfusion was made by a trauma team leader with more than 10 years of experience in the treatment of severely injured patients (senior anaesthetist/consultant). The treatment of the patient in the resuscitation room, including all the administered blood and coagulation products, was documented with the standard treatment form of the patient file. If resuscitation room care was aborted because the patient required immediate damage-control surgery, the blood products administered in the operating theatre were also recorded for this study. Survival and discharge rates were recorded 28 days after admission, and the injury severity score (ISS) was calculated on the basis of the diagnosis at the final hospital discharge.

### Data analysis and statistics

Based on the exclusion criteria and receipt of the written consent of the patient (or his legal representative), the prehospital and in-hospital datasets were merged and anonymised. Datasets with relevant missing parameters were excluded from the analyses.

The patients were divided into two groups for which descriptive data were obtained: patients who received and patients who did not receive transfusion of blood or coagulation products in the resuscitation room or during immediate surgery. The first step of the analysis was a bivariate comparison of the parameters. For this purpose, each parameter was examined for normal distribution (with the Shapiro–Wilk test), and then normally distributed data were analysed with the independent sample two-sided *t* test and data with a skewed distribution were analysed with the Mann–Whitney *U* test.

To assess prehospital lactate and base excess values as predictors of the need for early transfusion, a receiver operating characteristics (ROC) curve was created for both parameters and the area under the curve (AUROC) was determined. The optimal threshold values for lactate and base excess for this purpose were calculated using both Youden’s index and Euclidean distance.

In the next step, to develop a simple and pragmatic prediction model, the prehospital blood gas parameters lactate and base excess were supplemented with clinical parameters that are easy to measure. For this purpose, prehospital parameters were examined in a stepwise selection procedure for their independent association with the need for early transfusion. The parameters identified with this stepwise regression method were then used to create two new models one with lactate and one with base excess. AUROC values were calculated for these additional parameters and the two models.

All statistical analyses were performed using SPSS version 27 (IBM Corp., Armonk, NY, USA). The collected parameters are presented as mean ± standard deviation, and the 95% confidence interval (CI) is provided for the AUROC values. Statistical significance was assumed at *p* < 0.05.

## Results

**Fig. 1:**
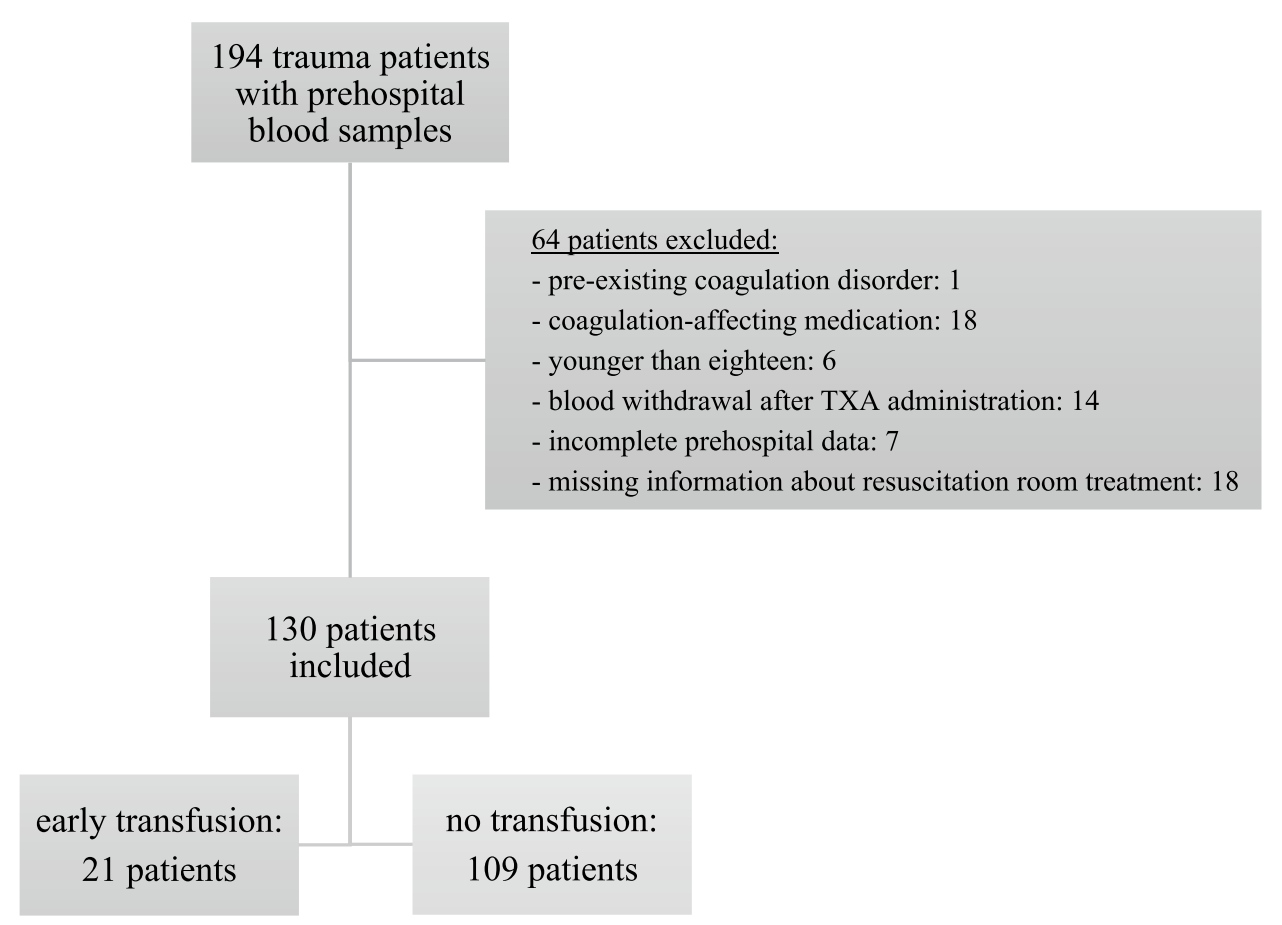
Patients flow chart. Presentation of included patients with prehospital blood samples and reasons for exclusion. *TXA* tranexamic acid

In the period from August 2015 to February 2018, prehospital blood samples were collected and data were documented for 194 trauma patients. As data for the exclusion criteria (one patient with existing coagulation disorder, 18 patients with use of coagulation-affecting medication, six patients younger than 18 years, 14 patients with blood withdrawal after tranexamic acid administration, and seven patients with incomplete prehospital data) were obtained only later, 46 patients had to be excluded from the analysis. In the case of 18 other patients, information about resuscitation room treatment and transfusions was not available. Thus, 130 of the initial 194 patients were subsequently included in the analysis (Fig. 1). For all the included patients, deferred written consent was provided by the patients themselves or their legal representatives.

Amongst the 130 patients studied, 75.4% were male, and the mean age was 45.4 ± 19.0 years (median 44.5 years; range 18–87). The mean ISS was 22.1 ± 16.8 (median 18; range, 3–75), and the 28-day mortality was 6.9%. Twenty-one patients required early blood transfusion, and the maximum number of transfusion units was 13 (including packed red blood cells, fresh frozen plasma, and platelets). The transfusion was started on average 28 min after hospital admission (time range 5–63 min after admission).

Table 1 shows the demographic data of the patients, the prehospital vital signs and infusion volumes, the results of the prehospital blood gas analysis, the initial blood gas analysis after hospital admission, the blood products early administered, and the 28-day mortality rate for the two groups of patients (those who required transfusion of blood and coagulation products and those who did not). Oxygen saturation and systolic blood pressure differed significantly between the two groups, but the initial heart rate did not. At the time of prehospital blood sampling, which was only a few minutes after trauma, all the listed parameters of the blood gas analysis were significantly different between the two groups. ISS and 28-day mortality were also significantly different between the two groups. Table 2 presents the injury severities and injury patterns, registered on the basis of the diagnoses at the final hospital discharge. The injury severity was higher in all body regions in those patients who received an early transfusion, and differed most in the body regions chest and abdominal or pelvic contents.

**Table 1:** Demographic data, prehospital vital signs, blood gas analysis data and blood transfusion data of trauma patients (*n* = 130) (Mean and standard deviation or percentage and number)

	Patients who did not receive blood transfusion (*n* = 109)	Patients who received blood transfusion (*n* = 21)	*p* value
Age [years]	47.6 ± 18.4	45.0 ± 19.2	0.56
Male sex, *n* (%)	83 (76%)	15 (71%)	0.65
Injury severity score	17.5 ± 11.6	45.5 ± 20.7	** < 0.001**
Pre-hospital data			
Initial heart rate [per min]	92.1 ± 17.6	91.6 ± 35.9	0.92
Initial saturation [%]	96.9 ± 12.9	84.0 ± 23.9	** < 0.001**
Initial systolic blood pressure [mmHg]	136.3 ± 25.6	88.8 ± 52.2	** < 0.001**
Crystalloids [ml]	736 ± 369	1110 ± 446	**0.001**
Colloids [ml]	60 ± 172	540 ± 377	** < 0.001**
pH	7.36 ± 0.08	7.20 ± 0.16	** < 0.001**
Lactate [mmol/l]	2.8 ± 1.2	4.5 ± 2.3	** < 0.001**
Base excess [mmol/l]	− 1.2 ± 3.3	− 7.4 ± 8.3	** < 0.001**
Haemoglobin [g/dl]	14.8 ± 2.0	11.3 ± 4.3	** < 0.001**
Mean time call to blood withdrawal [min]	26.1 ± 10.9	27.8 ± 10.6	0.53
Mean time call to hospital admission [min]	63.6 ± 15.0	58.8 ± 14.2	0.16
Suspected bleeding in the chest/abdomen/pelvis, *n* (%)	25 (23%)	16 (76%)	** < 0.001**
Cardio-circulatory instability (initial SBP < 100 mmHg), *n* (%)	8 (7%)	10 (48%)	** < 0.001**
Resuscitation room data			
pH	7.36 ± 0.06	7.24 ± 0.19	** < 0.001**
Lactate [mmol/l]	1.9 ± 1.0	3.5 ± 2.2	** < 0.001**
Base excess [mmol/l]	− 0.1 ± 3.4	− 6.1 ± 7.5	** < 0.001**
Haemoglobin [g/dl]	13.6 ± 1.9	9.2 ± 2.8	** < 0.001**
PRBC [units]	0	2.1 ± 2.2	
FFP [units]	0	3.1 ± 1.6	
Platelets [units]	0	0.1 ± 0.2	
28-day mortality rate, *n* (%)	3 (3%)	6 (29%)	** < 0.001**

The *p* values in bold indicate significant differences between the two groups

*SBP* systolic blood pressure, *PRBC* packed red blood cells, *FFP* fresh frozen plasma/freeze-dried plasma

**Table 2:** Injury severity and injury patterns of trauma patients (*n* = 130)

	Patients who did not receive blood transfusion (*n* = 109)	Patients who received blood transfusion (*n* = 21)	*p* value
Injury severity score	17.5 ± 11.6	45.5 ± 20.7	** < 0.001**
New injury severity score	22.0 ± 14.2	49.3 ± 19.3	** < 0.001**
Abbreviated Injury Scale			
Head/neck	1.6 ± 1.7	2.8 ± 2.4	**0.005**
Face	0.7 ± 1.0	1.9 ± 1.7	** < 0.001**
Chest	1.5 ± 1.7	3.2 ± 1.9	** < 0.001**
Abdominal or pelvic contents	0.8 ± 1.3	2.1 ± 1.6	** < 0.001**
Extremities or pelvic girdle	1.8 ± 1.3	2.5 ± 1.3	**0.02**
External	1.0 ± 0.9	2.1 ± 1.1	** < 0.001**

Classification of the body regions according to the calculation of the Injury Severity Score, the injury severity was documented on the basis of the diagnoses at the final hospital discharge according to the abbreviated injury scale (0–6). (Results presented as mean and standard deviation). The *p* values in bold indicate significant differences between the two groups

**Fig. 2:**
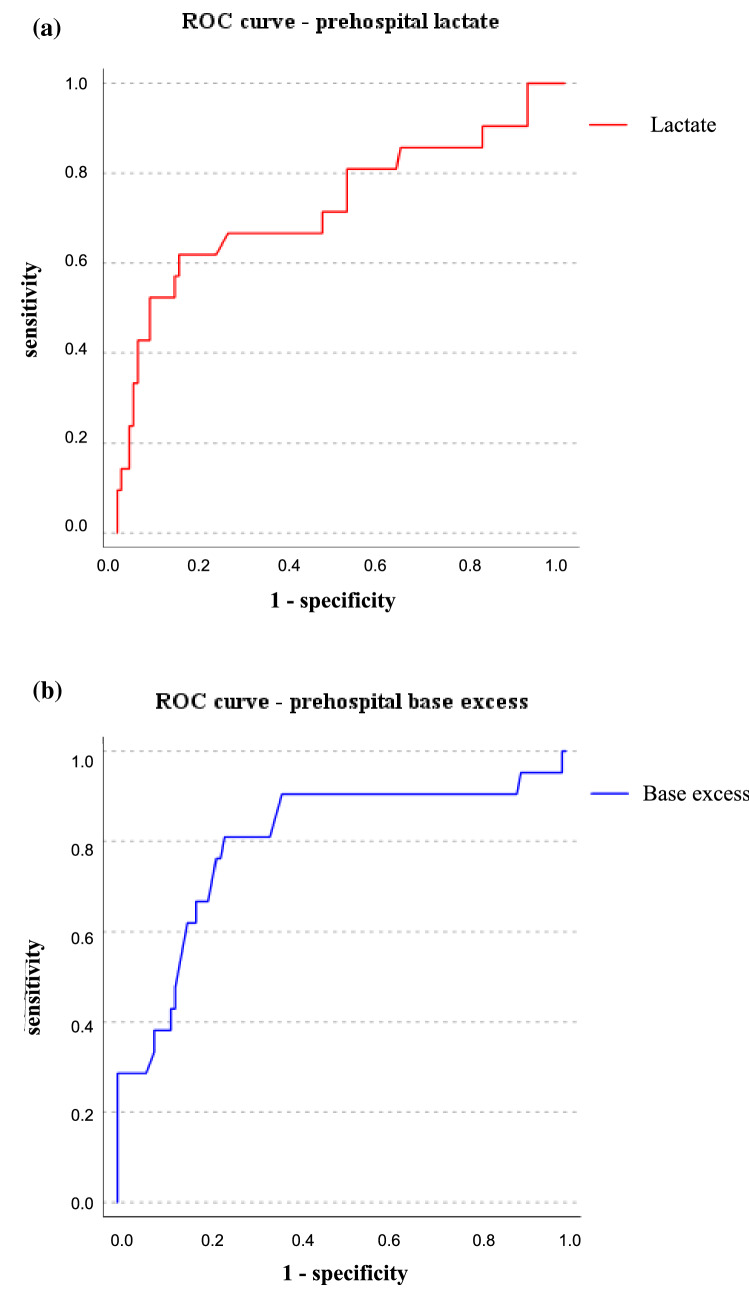
Lactate and base excess as transfusion predictors. Receiver Operating Characteristics (ROC) curve for prehospital lactate (**a**) and base excess (**b**) as a predictor of the need for early blood transfusion in 130 trauma patients. (area under the curve: **a** lactate 0.731, **b** base excess 0.798)

For the blood gas parameters lactate and base excess as predictors of the need for early blood transfusion, the ROC curves are shown in Fig. 2. The AUROC is 0.731 (95% CI 0.592–0.871) for lactate and 0.798 (95% CI 0.679–0.916) for base excess. The best cut-off values, calculated using the Youden’s index and Euclidean difference, are 4.1 mmol/l for lactate (sensitivity 0.65, specificity 0.86) and − 2.6 mmol/l for base excess (sensitivity 0.80, specificity 0.76). 100% specificity was achieved with lactate > 8.8 mmol/l and base excess < − 13.0 mmol/l.

To develop a simple and pragmatic prediction model, the next step was to examine other prehospital parameters for their association with early transfusion frequency. Based on a stepwise selection procedure, the variables prehospital cardiopulmonary resuscitation (CPR)/out-of-hospital cardiac arrest (*p* = 0.004), initial systolic blood pressure < 100 mmHg (*p* < 0.001) and suspected bleeding in the thorax/abdomen/pelvis (as determined by the EMS physician) (*p* = 0.001) seemed to have an influence on transfusion frequency. In contrast, age, gender, heart rate, oxygen saturation, and injury mechanism did not show any independent significant association with transfusion frequency (Table 3).

**Table 3:** Examined variables of the stepwise selection procedure for the development of an early transfusion prediction model in trauma patients (*n* = 130)

	Adjusted odds ratio	95% confidence interval	*p* value
Metric variables			
Age	1.02	0.99–1.06	0.52
Heart rate	1.02	0.99–1.07	0.17
Oxygen saturation	0.89	0.77–1.01	0.09
Initial systolic blood pressure	0.96	0.93–0.98	**0.01**
Binary variables			
Gender			0.32
Injury mechanism (blunt/penetrating)			0.31
Initial systolic blood pressure < 100 mmHg			** < 0.001**
Cardiopulmonary resuscitation/out-of-hospital cardiac arrest			**0.004**
Suspected bleeding chest/abdomen/pelvis			**0.001**

Metric variables are presented with adjusted odds ratios, 95% confidence interval and *p* value, binary variables only with *p* value. The *p* values in bold indicate significant differences between the two groups

The above results were merged to create a model for the prediction of the need for transfusion. For this purpose, the two blood gas parameters lactate and base excess were combined separately with the clinical parameters cardio-circulatory instability (initial systolic blood pressure < 100 mmHg) and suspected bleeding in the thorax/abdomen/pelvis. The parameter prehospital CPR was not included because no additional benefit in terms of the study objective was expected. The number of cases with CPR was only three, and furthermore, it can be assumed that a patient with trauma-associated out-of-hospital cardiac arrest has to be regarded as a critical patient.

**Fig. 3:**
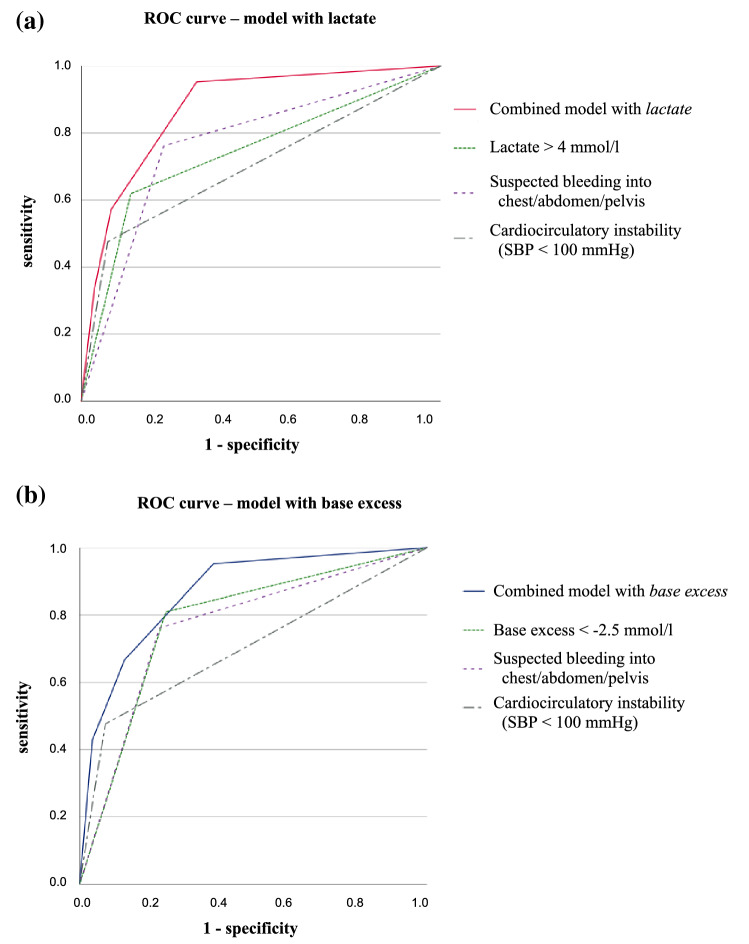
Combined prediction models for transfusion including lactate and base excess. Receiver Operating Characteristics (ROC) curves for predictors of the need for early blood transfusion in 130 trauma patients. The following parameters were tested individually and in combination to establish the models of scoring: **a** prehospital lactate > 4 mmol/l; suspected bleeding into the chest, abdomen and/or pelvis as determined by the EMS physician; and cardio-circulatory instability as indicated by systolic blood pressure (SBP) < 100 mmHg (area under the curve: combined model with lactate 0.871, lactate 0.741, suspected bleeding 0.766, cardio-circulatory instability 0.701). **b** prehospital base excess < − 2.5 mmol/l; suspected bleeding into the chest, abdomen and/or pelvis as determined by the EMS physician; cardio-circulatory instability as indicated by systolic blood pressure (SBP) < 100 mmHg (area under the curve: combined model with base excess 0.866, base excess 0.781, suspected bleeding 0.766, cardio-circulatory instability 0.701)

The threshold values of lactate and base excess were rounded to > 4.0 mmol/l and < − 2.5 mmol/l respectively. Figure 3 shows the ROC curves for the lactate and base excess model, respectively, by presenting the curves for individual parameters and their combination when the threshold values for all three parameters are crossed. For the lactate model (Fig. 3a), the AUROC was 0.871 (95% CI 0.794–0.949), and for the base excess model (Fig. 3b), the AUROC was 0.866 (95% CI 0.785–0.948).

## Discussion

The findings of this study show that lactate and base excess values determined from prehospital blood samples of severely injured patients were associated with the frequency of early in-hospital blood transfusions. By combining these values with relevant clinical parameters that were also easy to assess in the prehospital setting, this association was further strengthened to create a prediction model of the need for transfusion in the early stage after trauma.

Point-of-care measurements of the laboratory parameters lactate and base excess on admission to the resuscitation room have been widely studied in recent years, and their value as predictors of the need for blood transfusion or early damage-control surgery is well established [18, 19]. In the case of haemorrhagic shock, the values of these parameters may be early signs of peripheral ischaemia [20]. Furthermore, lactate and base excess are also associated with TIC, which in turn exacerbates haemorrhage [15, 21]. Accordingly, these values are included in some intra-hospital prediction scores for massive transfusion, such as the Trauma Associated Severe Haemorrhage (TASH) score [22]. However, if these parameters are only measured on arrival at the hospital, valuable time can be lost, especially in cases that require the activation of a massive transfusion protocol. The aim of this study, therefore, was to develop a simple tool to help EMS personnel at the scene of the incident to determine whether a blood transfusion might be necessary at an early stage. This information can then be used as a basis for initiating prehospital transfusion (if blood products are available) or activating the massive transfusion protocol of the receiving hospital. Against the background of the abovementioned correlations of lactate and base excess with TIC, these two prehospital point-of-care parameters were considered along with additional relevant clinical parameters. Based on our findings, we were able to confirm that lactate is an important prehospital measurement associated with the need for transfusion; additionally, we were able to demonstrate for the first time that the prehospital base excess value is also associated with the need for early transfusion. Comparable studies already exist for lactate, but not for base excess [23, 24]. In our study, the results of both blood gas parameters were comparable, as there was only a small difference in their AUROC values.

Our aim for the creation of this tool was to obtain the best possible result with the smallest number of parameters. This was important from the viewpoint of practicability, as the measurements need to be conducted within the least possible time in the prehospital setting. Therefore, in contrast to the work of Fukuma, in which six physiological parameters were included in the evaluation in addition to lactate, in this study, only the two strongest values were considered together with the two main blood gas parameters. Despite this simplification, the AUROC value reported by Fukuma (0.882) was similar to that calculated for our model (0.871) [24]. Additionally, in the present study, the lactate and base excess models showed similarity with regard to the prediction of early transfusion. Thus, lactate or base excess could be used, depending on their availability.

In addition to the abovementioned studies by Fukuma and Zadorozny, there have been other efforts in recent years to predict the need for transfusion with parameters that can be collected in the prehospital setting. One such study by Terceros-Almanza and colleagues attempted to use scoring systems that had already been validated for in-hospital use. They investigated six different scores for the prediction of massive transfusion in the prehospital setting, and some of these scores also included blood gas parameters [25]. However, there were various limitations with the application of these scoring systems. For example, the Assessment of Blood Consumption score [26] had an AUROC of only 0.68 for prehospital use, and the TASH score, which had an AUROC of 0.82, was not practical for prehospital use because it included seven variables with a score range of 0–29 [22]. The Emergency Transfusion Score emerged as the best one, but with six variables (some of which are measured with decimal numbers) and a range of 0–9.5, it also has limited applicability for prehospital use [27].

A score that was explicitly developed for prehospital prediction is the Early Blood Transfusion Needs Score [13]. This score includes five clinical parameters with an AUROC of 0.86, a cut-off value of > 5, a sensitivity of 0.83, and a specificity of 0.80. Importantly, this tool was evaluated in a large sample of over 24,000 patients. However, the authors used a retrospective design, and some of the data were obtained from in-hospital records. Additionally, the considerable difference in the weighting of the five parameters, with an overall range of − 4 to 17, could mean that it is not practical for making time-critical decisions in cases of severe injury.

Based on the advantages and disadvantages of the previously reported scoring tools discussed above, these are the merits of the scoring tool proposed in the present study. Our score only requires the measurement of three parameters that are equally weighted. Thus, it can be used to quickly identify patients with major trauma who are likely to require early blood transfusions. We have deliberately not calculated the prediction probabilities of the parameters, so as to provide EMS personnel with a decision support tool that can help them appropriately allocate severely limited blood products in the prehospital setting and identify patients who are likely to need transfusions in a timely manner. Compared to the studies described above, it therefore seems possible to make a prediction about early transfusion with fewer and easy to collect parameters with the model presented here, which would be roughly comparable in its statistical significance to the best scores published to date. However, this assessment must be viewed very cautiously; a detailed grading of this model should only be made after the currently ongoing validation study has been completed.

## Limitations

Several limitations have to be taken into account when evaluating the present results. Due to the monocentric, prospective study design, the number of cases is relatively small, and the statistical results should, therefore, be interpreted with caution. Unfortunately, 18 patients had to be excluded because the receiving hospital was not one of the two level I trauma centres in Ulm. However, this restriction in the study design was necessary to ensure that the patients were reliably informed about their participation in the study and that complete data could be obtained about the clinical treatment of the patients.

Due to the small number of cases, the simple score developed in this study should be used very cautiously for now. This scoring tool has not been validated yet, and especially there was no external validation. The small sample size would have limited the results of derivation and validation analysis within one and the same study. However, a prospective follow-up study is currently being conducted (TIC-DETECT, DRKS registration no. 00015886). This ongoing study includes a larger sample size of severely injured patients to validate the observed correlations reported here.

We did not determine the exact prediction probabilities of the score or the intermediate range of the lactate and base excess models. The statistical analysis would not have been robust enough due to the small number of cases. Therefore, it was also not possible to consider weightings of the individual parameters. However, this was deliberately omitted because the aim of developing the tool was to keep it as simple and practicable as possible for the prehospital setting.

## Conclusion

The lactate and base excess values of severely injured patients at the place of incident have been reported to be associated with the need for early blood transfusion in the resuscitation room or during immediate surgery. With the model proposed in the present study, the predictability of these parameters can be improved with the addition of other clinical parameters that are easy to collect in the prehospital setting. If this association can be confirmed through a validation study, this model could be used as a decision-support tool for prehospital blood transfusions. Furthermore, by informing the receiving hospital at an early stage, in-hospital massive transfusion protocols could be initiated on time.

## Declarations

### Presentation

This study is part of the doctoral thesis of Julia Riedel (Medical Faculty of Ulm University).
